# Predictors of Satisfactory Surgical Outcome in Idiopathic Normal Pressure Hydrocephalus (Review)

**DOI:** 10.17691/stm2024.16.2.07

**Published:** 2024-04-27

**Authors:** A.V. Stanishevskiy, G.V. Gavrilov, M.N. Radkov, B.G. Adlejba, D.V. Svistov

**Affiliations:** Neurosurgeon, PhD Student, Department of Neurosurgery; Military Medical Academy named after S.M. Kirov, 6 Academician Lebedev St., Saint Petersburg, 194044, Russia; MD, DSc, Associate Professor, Lecturer, Department of Neurosurgery; Military Medical Academy named after S.M. Kirov, 6 Academician Lebedev St., Saint Petersburg, 194044, Russia; Head of the Department of Neurosurgery No.2; Academician I.P. Pavlov First St. Petersburg State Medical University, 6–8 L’va Tolstogo St., Saint Petersburg, 197022, Russia; Neurosurgeon, Department of Neurosurgery; Military Medical Academy named after S.M. Kirov, 6 Academician Lebedev St., Saint Petersburg, 194044, Russia; Neurosurgeon, PhD Student, Department of Neurosurgery; Military Medical Academy named after S.M. Kirov, 6 Academician Lebedev St., Saint Petersburg, 194044, Russia; MD, PhD, Associate Professor, Head of the Department of Neurosurgery; Military Medical Academy named after S.M. Kirov, 6 Academician Lebedev St., Saint Petersburg, 194044, Russia

**Keywords:** idiopathic normal pressure hydrocephalus, predictor, neuroimaging, tap test, invasive diagnosis

## Abstract

Idiopathic normal pressure hydrocephalus is a widespread neurodegenerative disease of the elderly. If not treated surgically early, it results in a severe decrease in quality of life and disability. According to current clinical Russian and foreign guidelines the candidates for CSF shunting procedures are selected based on the results of invasive tests, though treatment outcomes are not always optimal. At the same time, in the last decade there have been published a number of studies on promising noninvasive diagnosis and prognosis of the surgical treatment of idiopathic normal pressure hydrocephalus based on neuroimaging findings.

**The aim of the present systematic review** is to demonstrate the most promising imaging predictors of satisfactory outcomes of CSF shunting procedures in patients with idiopathic normal pressure hydrocephalus based on published literature data.

## Introduction

Idiopathic normal pressure hydrocephalus (iNPH, Hakim–Adams syndrome) is a steadily progressive neurodegenerative disease, as a rule occurring in patients over 60, and characterized by an extension of CSF-containing brain spaces against the background of normal cerebrospinal fluid pressure, and represented by a triad of symptoms: impaired gait, cognitive sphere and pelvic organs functioning (Hakim–Adams triad). A unique characteristic of iNPH is possible complete or partial regress of symptoms in case of early surgical treatment — cerebrospinal fluid (CSF) shunting procedures [1]. However, according to large series of observations [2–7], patients’ improvement after CSF shunting procedures starts on average in 70.4% cases. No dynamics in patient’s state after CSF shunting procedures can be related to both: iNPH misdiagnosis (iNPH can be easily taken for other disease with similar presentation, e.g. Alzheimer’s disease, Parkinson’s disease, Binswanger’s disease, frontotemporal dementia, etc.), as well as with the surgery performed in the period of the disease when the symptoms are irreversible [8–11]. However, CSF shunting procedures are risk-bearing of complications including severe and life-threatening ones (Table 1).

**T a b l e 1:** CSF shunting procedure efficiency in idiopathic normal pressure hydrocephaly (%)

Reference	CSF shunting procedure efficiency	Complication risk	Fatality rate
Hebb and Cusimano, 2001 [2]	59	38	6
Toma et al., 2013 [3]	71	8.2	1
Eide and Sorteberg, 2016 [4]	90	3.5	1.3
Giordan et al., 2018 [5]	75	9	<2
Hong et al., 2018 [6]	54.8	32	3.2
Greuter et al., 2022 [7]	72.8	51.1	Not evaluated

According to current clinical recommendations [1, 12, 13], a decision on performing CSF shunting procedures is taken based on invasive diagnostic techniques. A systematic review by Thavarajasingam et al. [14] showed that among invasive techniques used for CSF shunting procedure outcome prognosis, the most effective (in decreasing order) ones are: intra-cranial pressure (ICP) monitoring using a parenchymatous sensor, prolonged external lumbar drainage of cerebrospinal fluid, an infusion-loading test and a tap test. The mentioned diagnostic procedures enhance the likelihood of CSF shunting procedure favorable outcome; however, do not ensure the postoperative neurological deficit regress. Moreover, the procedures are associated with the necessity of admission to a special hospital to carry out surgeries — lumbar puncture, external lumbar drainage or ICP sensor. Therefore, according to the polling of specialists involved in the disease treatment, the development of a save noninvasive technique for iNPH diagnosis is one of priority tasks for clinical research [15]. At the same time, a detailed volumetric analysis of brain structures and compartments based on MRI is indicative of high prognostic efficiency of the method in revealing the patients, in whom CSF shunting procedures are to result in positive dynamics of symptoms [16]. Similar findings were also obtained in morphometric assessment of grey matter in patients with hydrocephaly [17]. Thus, there are there are objective grounds to believe that the brain morphology changes revealed by neuroimaging in iNPH can serve as predictors of CSF shunting procedure favorable outcome.

**The aim of the present review** is to analyze the literature data on the most valuable imaging symptoms of idiopathic normal pressure hydrocephalus in relation to CSF shunting procedure prognosis.

## Materials and Methods

The present systematic review is carried out in accordance with PRISMA (Preferred Reporting Items for Systematic Reviews and Meta-Analyses) criteria [18]. The literature was searched in databases: RSCI, PubMed/MEDLINE, Scopus, Web of Science, as well as using a searching system Google Scholar. Furthermore, there were selected the publications referring to the articles found as cited by, or those have similar descriptions (similar articles). After excluding the works, which are doubled in several sources, the bibliographic data and abstracts of the rest articles were studied concerning inclusion criteria match.

The study is a systematic review requiring no Ethics Committee approval.

**Figure 1.**
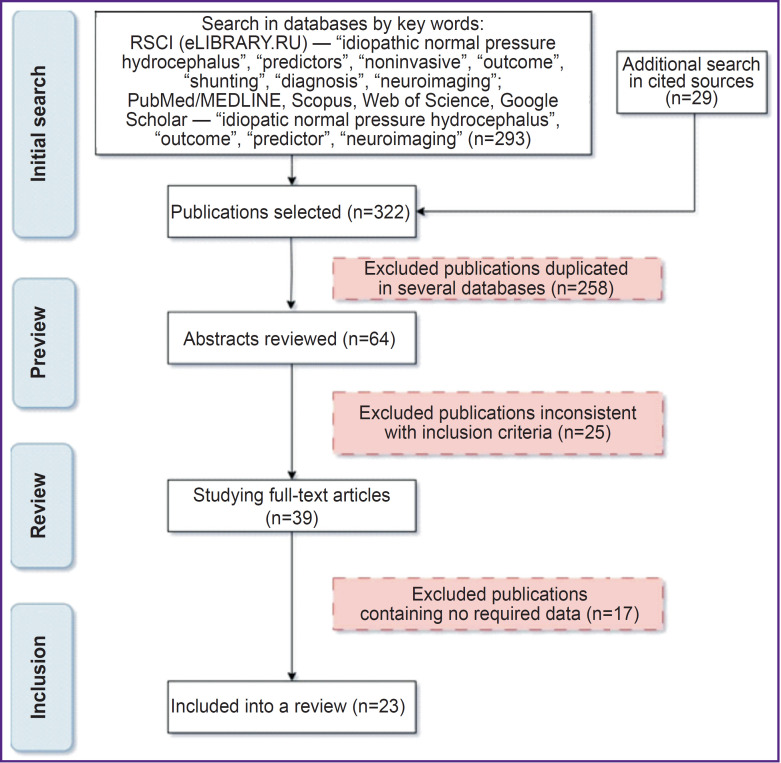
Methods for literature search for a systematic review

The review includes the works carried out not until 2013, in Russian and English, having access to a full text (or an abstract with all necessary data), which describe the investigations devoted to detection of imaging predictors of CSF shunting procedure favorable outcome in iNPH patients. Figure 1 demonstrates the stages of literature search.

## Results

The review includes 23 [6, 9, 16, 19–38] studies (22 original works and 1 meta-analysis). The analyzed predictors of CSF shunting procedure outcome were admitted reliable in 14 publications, while 9 ones revealed no statistically significant differences in surgery outcomes between the patients with and without the studied symptoms. Table 2 represents the imaging predictors of CSF shunting procedure outcomes stated through literature data analysis and ranged by the number of studies found them.

**T a b l e 2:** Rating of CSF shunting procedure outcome predictors revealed in the review

Imaging symptom	Number of publications recognizing it as a CSF shunting procedure outcome predictor	Number of publications not confirmed it status of a CSF shunting procedure outcome predictor
Hydrocephaly syndrome with irregular extension of CSF spaces (DESH syndrome)	4	7
Quantitative assessment of DESH syndrome (DESH score)	2	1
Callosal angle	3	7
Anterior modification of callosal angle	1	—
Compression of convexital subarachnoid space in the vertex area	2	3
Evans index	1	9
Score according to radiological scale of iNPH diagnosis (iNPH Radscale)	1	1
Periventricular changes of MR signal	1	6
Width of temporal horns of the lateral ventricles	1	3
Local extension of convexital hemispheric sulci	1	4
Extension of Sylvian fissures	1	4
Volumetric analysis of brain structures	1	—
Number of lacunar infarction foci	1	—
Complex analysis of imaging data	1	—

MR-signal changes in the white brain matter, cingulate gyrus size, ventricle III diameter and ventricle IV size, great longitudinal fissure extension, CSF movement artefacts, hippocampal atrophy signs, the vertical size of the lateral ventricles and their roof bulging, SILVER-index value according to literature data cannot be considered as imaging predictors of CSF shunting procedure outcome in iNPH patients. Table 3 demonstrates the generalized systematic review findings.

**T a b l e 3:** Brief description of study findings included in a systemic review

Reference	Number of iNPH patients	Imaging parameters under study	Conclusions
***Unspecified predictors of CSF shunting procedure outcome***			
Chen et al., 2022 [19]	47	Evans index Score according to iNPH Radscale DESH syndrome Callosal angle	The values of the studied parameters had no significant difference in the groups with positive response to CSF shunting procedure and those without significant changes
Snöbohm et al., 2022 [20]	253	Periventricular changes of MR signal MR signal changes in the white matter	The specified changes are not CSF shunting procedure outcome predictors
Laticevschi et al., 2021 [21]	179	Score according to iNPH Radscale	Total score of the scale has no correlation with tap test results
Skalický et al., 2021 [22]	32	Cingulate gyrus size Callosal angle DESH syndrome (quantitative assessment)	The studied parameters are not reliable CSF shunting procedure outcome predictors
Agerskov et al., 2019 [23]	168	Evans index Width of temporal horns of the lateral ventricles Callosal angle Ventricle III diameter Ventricle IV size Local extension of convexital hemispheric sulci Compression of convexital subarachnoid space in the vertex area Extension of Sylvian fissures DESH syndrome CSF motion artifacts Periventricular changes of MR signal MR signal changes in the white matter	The parameters were evaluated separately between the groups of patients with a positive response to CSF shunting procedure (n=115) and without (n=53). No significant differences were revealed in no parameters under study
Ahmed et al., 2018 [24]	162	DESH syndrome	Improvement was stated in both patients’ groups — with and without DESH syndrome, with no significant differences
Benedetto et al., 2017 [25]	29	Evans index SILVER index value	There was suggested SILVER index (the ratio of Sylvian fissure area and convexital subarachnoid space on one coronal section). Neither Evans index nor SILVER index can be considered as CSF shunting procedure outcome predictors
Craven et al., 2016 [26]	103	DESH syndrome	Low value of negative prognostic value for DESH syndrome does not make it possible to use it as an independent predictor of CSF shunting procedure outcome
Kojoukhova et al., 2015 [27]	229	Evans index DESH syndrome MR signal changes in the white matter CSF motion artifacts Extension of temporal horns of the lateral ventricles Local extension of convexital hemispheric sulci Callosal angle	DESH syndrome is associated with iNPH diagnosis, however, no one from the studied symptoms is not a reliable CSF shunting procedure outcome predictor
***Specified predictors of CSF shunting procedure outcome***			
Thavarajasingam et al., 2023 [28]	Meta-analysis of 28 studies	DESH syndrome Callosal angle Periventricular changes of MR signal	Callosal angle value and periventricular changes of MR signal are CSF shunting procedure outcome predictor, however, can be used as a whole with other diagnostic criteria
Johannsson et al., 2022 [29]	55	Evans index Quantitative assessment of DESH syndrome Ventricle III diameter Extension of Sylvian fissures Callosal angle Local extension of convexital hemispheric sulci Compression of convexital subarachnoid space in the vertex area	Compression of convexital subarachnoid space in the vertex area, extension of Sylvian fissures, local extension of convexital hemispheric sulci and quantitative assessment of DESH syndrome (DESH score) is a reliable CSF shunting procedure outcome predictor
Kimura et al., 2021 [9]	154	DESH syndrome	There was revealed strong correlation between DESH syndrome and the patient’s improvement a year after CSF shunting procedure
Mantovani et al., 2021 [30]	47	Callosal angle Anterior callosal angle	Assessment of the anterior callosal angle (values under 112°) is a reliable CSF shunting procedure outcome predictor
Subramanian et al., 2021 [31]	37	Evans index Width of temporal horns of the lateral ventricles Ventricle III diameter Callosal angle Local bulging of the lateral ventricles roof Corpus callosum thickness Extension of Sylvian fissures Compression of convexital subarachnoid space in the vertex area	High values (the border was not determined) of Evans index are the predictor of improved cognitive functions after CSF shunting procedures. Callosal angle and DESH syndrome are not CSF shunting procedure outcome predictors
Wu et al., 2021 [16]	145	Volumetric analysis of brain structures	Some brain morphologic parameters can serve as CSF shunting procedures outcome predictor
Wolfsegger et al., 2021 [32]	21	iNPH Radscale	There were determined the scale value limit (7.5 points) discriminating patients by CSF shunting procedure response
Gavrilov et al., 2019 [33]	213	Evans index Ventricle III diameter Width of temporal horns of the lateral ventricles DESH syndrome Local extension of convexital hemispheric sulci Callosal angle Periventricular changes MR signal changes in the white matter Extension of periventricular spaces	There were demonstrated the advantages of a complex assessment of MRI sign in iNPH differential diagnosis
Grahnke et al., 2018 [34]	73	Evans index Periventricular changes of MR signal Callosal angle Height of the lateral ventricles	Callosal angle is a CSF shunting procedure outcome predictor
Hong et al., 2018 [6]	31	Evans index Periventricular changes of MR signal MR signal changes in the white matter Number of lacunar infarction foci Hippocampal atrophy DESH syndrome Callosal angle	Among the studied factors only DESH syndrome and the number of lacunar infarction foci significantly differed for patients’ groups with and without effect from CSF shunting procedures
Shinoda et al., 2017 [35]	50	DESH syndrome (quantitative assessment)	Quantitative assessment of DESH syndrome is a reliable CSF shunting procedure outcome predictor
Garcia-Armengol et al., 2016 [36]	89	DESH syndrome	Prognostic characteristics of DESH syndrome and intracranial pressure monitoring are comparable
Narita et al., 2016 [37]	60	Evans index Compression of convexital subarachnoid space in the vertex area Extension of Sylvian fissures Callosal angle Local extension of convexital hemispheric sulci Local bulging of the lateral ventricles roof Periventricular changes of MR signal MR signal changes in the white matter	Compression of convexital subarachnoid space in the vertex area is a reliable CSF shunting procedure outcome predictor
Virhammar et al., 2014 [38]	108	Evans index Width of temporal horns of the lateral ventricles Ventricle III diameter Callosal angle Local bulging of the lateral ventricles roof Extension of Sylvian fissures Compression of convexital subarachnoid spaces in the vertex area Local extension of convexital hemispheric sulci DESH syndrome Periventricular changes of MR signal MR signal changes in the white matter	Callosal angle, width of temporal horns of the lateral ventricles and DESH syndrome are CSF shunting procedure outcome predictors

N o t e: iNTH — idiopathic normal pressure hydrocephalus.

## Discussion

The idea of limiting or complete refusal of invasive studies for CSF shunting procedure outcome prognosis is dictated by the following reasons.

Firstly, their use is risky, since the complication rate of external lumbar CSF drainage reaches 8.2%, and among them 3% are severe complications (subdural hematomas, infectious complications, etc.) [26].

Secondly, a positive result of the studies does not ensure any improvement in patient’s CSF shunting procedure postoperative state, while a negative result does not always enable to rule out iNPH. For instance, the presence of marked degenerative and dystrophic spinal changes in elderly patients can result in false results of tap-test, infusion-loading test and prolonged external lumbar CSF drainage [39].

Thirdly, the use of invasive studies requires inpatient treatment. Based on the analysis of cost-effectiveness and complication risks when using invasive iNPH diagnostic techniques, Eide et al. [40] indicated the necessity to search for other ways of CSF shunting procedure outcome prognosis.

Fourthly, the necessity to perform an invasive procedure for diagnostic purposes frequently decreases medication adherence and increases the time interval between the onset of iNPH symptoms and CSF shunting procedures. Moreover, some researchers prove conclusively that this parameter has a great influence on treatment result [9, 10, 41]. The patients operated on within the first 3 months after iNPH diagnosis is made appear to have the best outcome [10]. This fact also indicates the necessity of early disease detection and reduction of decision making period of CSF shunting procedures.

The third edition of Guidelines for management of iNPH by the Japanese Society of Normal Pressure Hydrocephalus [13] for the first time has assigned the possibility to make an iNPH diagnosis without invasive studies: DESH syndrome is recognized to be a diagnostic criterion equally ranking with a spinal tap test and prolonged lumbar CSF drainage.

Apart from DESH syndrome, various neuroimaging criteria were studied concerning CSF shunting procedure outcome prognosis. They are accepted to be distinguished into morphological and physiological [42]. Morphological symptoms include the changes of brain structures and spatial relationship of its parts (Evans index, DESH syndrome, callosal angle variation, periventricular changes, irregular extension of convexital subarachnoid spaces, the extension of temporal horns of lateral ventricles, etc.) revealed, as a rule, by routine procedures methods — CT and standard sequences of brain MRI. Physiological symptoms include the changes of the parameters determined by complex specialized techniques — CT- and MR-perfusion, MR–CSF dynamics, lymph MRI, as well as the changes in blood flow parameters in cerebral arteries and veins [43] and others.

According to the findings of meta-analysis performed by Thavarajasingam et al. [28], among the analyzed radiological symptoms (DESH syndrome, callosal angle, periventricular changes, cerebral blood flow, and cisternography findings) only callosal angle value and periventricular changes significantly differed between patients groups with positive and negative CSF shunting procedure outcome, except that the prognostic value of the parameters is not high. The authors marked the necessity to study the capabilities of a complex evaluation of neuroimaging symptoms for CSF shunting procedure outcome prognosis. A systematic review by Carlsen et al. [42] based on the analyzed 27 publications showed similar data. The findings of the present review correspond to the data: Table 2 and Table 3 demonstrate that just few imaging symptoms under study significantly differ in a group of patients with positive CSF shunting procedure outcome.

In an effort to improve efficiency of CSF shunting procedure outcome prognosis some authors tried to unite some neuroimaging iNPH symptoms into systems and scales. Ishii et al. [44] were the first who had an attempt to evaluate neuroimaging parameters for iNPH differential diagnosis. As follows from the correlation of Evans index values and colossal angle, the authors to a high precision succeeded in differentiating patients with iNPH, Alzheimer’s disease and those from a control group [44]. The best known scale for iNPH diagnosis by imaging data is iNPH Radscale including 7 parameters evaluated on brain computed tomograms [45]. The scale peculiarities are the assessment of morphological changes of brain structures according to CT, as well as no analysis of “contribution” of each parameter into iNPH diagnosis. It is just the thing related to the criticism by some researchers [19, 21, 33].

**Figure 2.**
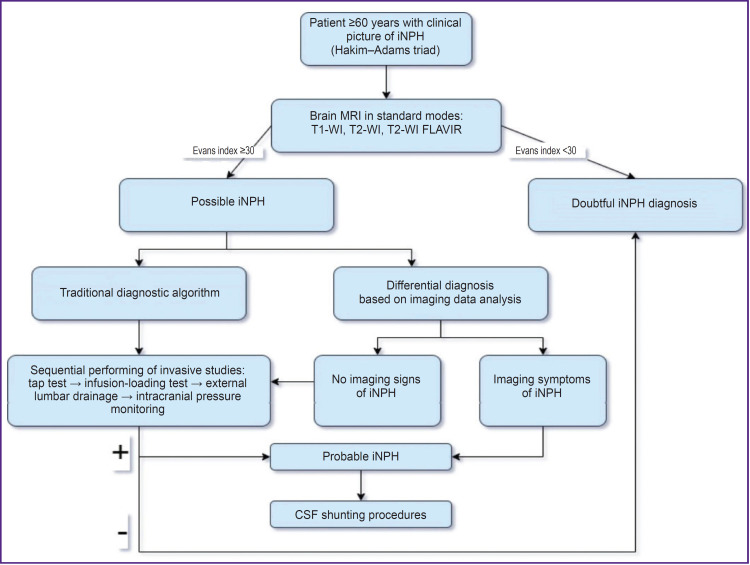
Suggested diagnostic algorithm of idiopathic normal pressure hydrocephalus (iNPH) and selection of candidates for CSF shunting procedures, based on a systemic review

Gavrilov et al. [33] had an attempt to group the most informative neuroimaging iNPH predictors and unify them into differential diagnostic system using statistical methods of discriminant analysis and classification. The developed system enables to a high precision differentiate between iNPH and the diseases having similar presentation based on a complex assessment of MRI data. Further studies in this sphere aim at assembling to a similar system the predictors of CSF shunting procedure positive outcomes and on the obtained base forming an advanced algorithm of taking clinical decision limiting or completely excluding invasive procedures. Based on the data analysis made in systematic review the authors suggest the following iNPH diagnostic algorithm and candidates’ selection to perform CSF shunting procedures, which unites currently available knowledge (Figure 2).

The next stage of improving iNPH diagnosis and the selection of candidates for CSF shunting procedures according to neuroimaging data can be the implementation of systems using artificial intelligence and computer-aided learning algorithms [46].

## Conclusion

The analysis of the present systemic review established 12 predictors of the positive CSF shunting procedure outcome; the predictors proved their efficiency in the course of clinical studies. Further efforts should aim at uniting the revealed predictors into a system for CSF shunting procedure outcome prognosis. Establishing such system will enable to restrict or completely exclude the necessity to use invasive techniques.
